# Assessing Community Acceptance of Maternal Immunisation in Rural KwaZulu-Natal, South Africa: A Qualitative Investigation

**DOI:** 10.3390/vaccines10030415

**Published:** 2022-03-10

**Authors:** Rujeko Samanthia Chimukuche, Nothando Ngwenya, Janet Seeley, Petronella Samukelisiwe Nxumalo, Zama Pinky Nxumalo, Motlatso Godongwana, Nomasonto Radebe, Nellie Myburgh, Sunday A. Adedini, Clare Cutland

**Affiliations:** 1 Social Science Department, Africa Health Research Institute, Mtubatuba 3935, South Africa; nothando.ngwenya@ahri.org (N.N.); janet.seeley@lshtm.ac.uk (J.S.); samukelisiwe.nxumalo@ahri.org (P.S.N.); zama.nxumalo@ahri.org (Z.P.N.); 2 School of Nursing and Public Health, University of KwaZulu-Natal, Durban 4041, South Africa; 3 Department of Global Health and Development, London School of Tropical Hygiene and Medicine, London WC1H 9SH, UK; 4 Programme in Demography and Population Studies, Schools of Public Health and Social Sciences, University of the Witwatersrand, Johannesburg 2193, South Africa; motlatsog@genesis-analytics.com (M.G.); sunday.adedini@fuoye.edu.ng (S.A.A.); 5 Medical Research Council: Wits Vaccines and Infectious Diseases Analytics Unit, Faculty of Health Sciences, University of the Witwatersrand, Johannesburg 2193, South Africa; nomasonto.radebe@wits-vida.org (N.R.); nellie.myburgh@wits-vida.org (N.M.); clare.cutland@wits.ac.za (C.C.); 6 African Leadership in Vaccinology Expertise (Alive), Faculty of Health Sciences, University of the Witwatersrand, Johannesburg 2050, South Africa; 7 Demography and Social Statistics Department, Federal University, Oye-Ekiti 371104, Nigeria

**Keywords:** maternal immunisation, maternal healthcare, tetanus toxoid, vaccine uptake

## Abstract

Despite the significant benefits of maternal immunisation, uptake remains low in many parts of the world. In this qualitative study, we aimed to assess the factors that influence pregnant women’s decision to engage with maternal immunisation in rural KwaZulu-Natal, South Africa. We conducted in-depth interviews with a total of 28 purposively sampled pregnant women and key informants using semi-structured topic guides. Data analysis was conducted using a modified Health Belief Model framework that included constructs of barriers to action, modifying factors of cue to action and perceived social norms. The findings show that traditional customs and institutional barriers such as low-quality health service delivery, long queues, and distance to the health facilities, immunisation vaccine stockouts and low levels of maternal knowledge influence the choice and decision to engage with maternal immunisation. Understanding health-related behaviours and addressing barriers to care is important in facilitating vaccination uptake. This study contributes to the understanding of maternal immunisation uptake in low-resource settings.

## 1. Introduction

Maternal immunisation acceptance and uptake is influenced by several external factors including time, place and type of vaccine [1]. In low- to middle-income countries (LMICs), low levels of education, a lack of maternal immunisation vaccine knowledge and challenges in health service delivery also hinder uptake [1]. The World Health Organisation (WHO) recommends maternal vaccination against tetanus, pertussis and influenza, as well as other diseases such as pneumococcus [2,3]. In many LMIC, including South Africa, tetanus toxoid immunisation is recommended for pregnant women to prevent neonatal tetanus [4,5]. According to the WHO, a gradual decline in the uptake of the third dose of diphtheria–tetanus–pertussis-containing vaccines (DTP3) has been noted, from 85% in 2014 to 77% in 2019 [6].

In the last two decades, South Africa has made significant progress in improving maternal health with the maternal mortality ratio at 119 per 100,000 live births. This has mainly been due to the increase in national rollout programmes that have led to improved antenatal care coverage and maternal health service delivery [7,8,9,10]. Notably, health workers were mandated to deliver maternal immunisation to pregnant women from the Expanded Programme on Immunisation implemented across all provinces in South Africa (EPI-SA) [4,11]. As a result, maternal healthcare service utilisation has increased significantly, from 83.4% in 1998 to 96.7% in 2016 [9]. In uMkhanyakude District, northern KwaZulu-Natal province, there has been an improvement in maternal immunisation uptake over the past five years. Remarkably, a rate of 72.5% of antenatal visits to health facilities has been recorded before 20 weeks [12]. Despite this improvement, suboptimal antenatal and maternal care continues in the district, the root causes of which are attributed to the failure to adhere to maternal health guidelines and harmful traditional practices [12].

In this paper, we describe the perceptions of maternal immunisation among pregnant women and other key informants in northern KwaZulu-Natal and the influence this has on maternal acceptance of the tetanus toxoid vaccine, and what might be done to increase vaccine confidence and improve uptake.

## 2. Materials and Methods

This study was conducted in rural uMkhanyakude district of northern KwaZulu-Natal. This district is one of the poorest in South Africa, with 98% of the population living in rural homesteads; 22% have access to safe water; only 10% of households are within a short distance, approximately 4.72 km, of a health clinic [12,13]. The district is situated within the Mpukunyoni Tribal Authority and the community is guided by tribal laws, customs and traditional structures. The sub-study results on which this paper draws are part of a larger study, assessing community acceptance and health facility preparedness for the implementation of maternal immunisation programs in urban and rural South Africa funded through IMPRINT—Immunising Pregnant Women and Infants Network. The overall IMPRINT study aimed to understand the knowledge, attitudes and acceptability of maternal immunisation amongst pregnant and non-pregnant women, healthcare providers and community members in rural and urban South Africa [14].

### 2.1. Data Collection

The study design was exploratory, and we used qualitative data collection methods that included in-depth interviews and focus group discussions. Individual interviews were conducted with 28 participants. Six of the participants were interviewed via the telephone later in June 2020 because of the COVID-19 non-pharmacological measures in place at that time. One focus group discussion was conducted with five pregnant women of different age groups. Table 1 gives a description of the study participants. The sample comprised women who were unemployed, school dropouts and students. Topic guides were translated into the local language, isiZulu, and back translated into English. After gaining informed consent, the interviews were conducted in IsiZulu from December 2019 to June 2020. All data collection activities were digitally recorded, transcribed verbatim and then translated to English. We provide the topic guides we used in Supplementary Materials (S1–S5).

Interviews were conducted by trained field workers in private settings where the participants felt comfortable. Interviews lasted approximately forty-five minutes to an hour. Interview summaries were written by the fieldworkers immediately after each interview to provide an overview of the interview and the main points raised and to complement the transcription, which took longer to produce. Debriefings between the lead researchers and the fieldworkers were conducted after each interview. Data quality checks were conducted by the facilitators to ensure the completeness and accuracy of transcripts. Participants were given identification numbers; these are used in the presentation of our results to allow readers to distinguish between quotes from different people.

### 2.2. Data Analysis and Interpretation

Thematic content analysis was conducted manually by two authors (RSC) and (NN), who are experienced social scientists. Data were managed using a framework analysis approach. The theoretical framing of the Health Belief Model (HBM) was used as a guide to identify and group emerging themes related to the acceptability of maternal immunisation. Themes related to HBM constructs were identified through coding and data were copied and pasted into excel sheets according to thematic areas. Indexing (coding) and charting (copying and pasting data according to thematic areas) were carried out simultaneously. 

### 2.3. Theoretical Framework

The Health Belief Model (HBM) is one of the most widely used theoretical frameworks for understanding health behaviour [15,16]. This model is used to assess intra-personal factors, including risk-related beliefs that may influence individuals’ health decision making [17]. 

The HBM conceptual framework comprises six constructs that predict health behaviours, namely, perceived susceptibility, perceived severity, benefits to action, barriers to action, cue to action and self-efficacy [18,19]. The HBM focuses on health behaviour and perceptions towards an illness and prevention.

**Figure 1:**
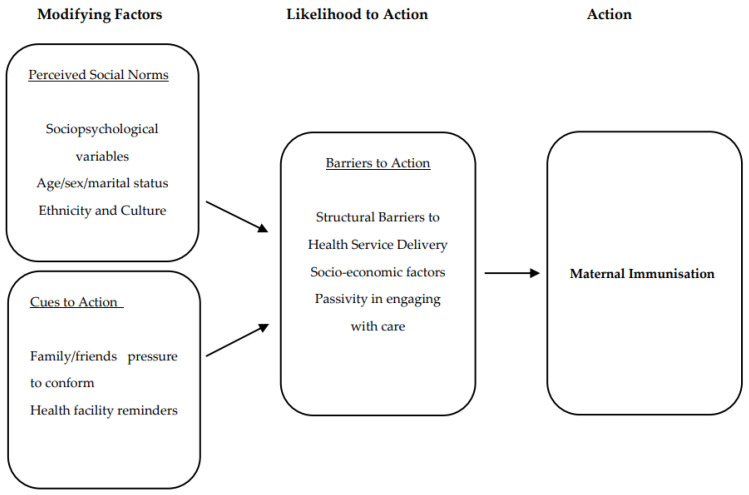
The Health Belief Model, adapted from Rosenstock et al. (1974).

For the purposes of this analysis, we used a modified HBM as illustrated in Figure 1. The HBM analytical framework was used as a foundation for our data analysis. The HBM states that people will take action to prevent illness if they regard themselves as susceptible to a disease (perceived susceptibility) and if they believe it would have potentially serious consequences (perceived severity) [19]. In preparing our coding framework, with the HBM as a basis, we observed additional factors that motivated people to disengage in preventive health behaviours beyond those originally specified by the HBM. Social norms have been significant predictors of health behaviours in our study setting and can predict health behaviours towards interventions [20], while susceptibility and the perceived severity of disease were seldom mentioned, because of limited awareness among participants on the diseases that maternal vaccination might prevent. 

Social norms often relate to perceived social pressure to engage or not engage in specific behaviours [21]. Attitudes and cultural beliefs shape individuals’ health behaviour and are strong motivators of behavioural change [22]. Taking these factors into account, we modified the HBM framework to include constructs of barriers to action, perceived social norms and cues to action. 

## 3. Results

A total of 22 face-to-face in-depth interviews, six telephone interviews and one focus group with pregnant women of different ages and key informants were conducted. The pregnant women were aged between 18 and 35 years. Six key informants were traditional and church healers aged between 30 and 71 years. Four key informants were maternity staff specialising in advanced midwifery and one was a retired community midwife aged 79 years. 

### 3.1. Modifying Factors

#### 3.1.1. Sociopsychological Variables

Caregivers and the partners of young pregnant women (aged 18–35 years) were an important influence on the woman’s health and her likelihood of accessing care. Pregnant women may disengage with maternal care because of their partners belief about appropriate maternal care. Partners of pregnant women may negatively influence engagement with maternal care, especially if the women are financially dependent on them.

“…*I don’t know why, but there are others [men] who refuse [to allow] their partners to go to the clinic. Others [some men] don’t like the fact that their partners will be pursued by other men on the road. Others just don’t want to give them money…for transport*…”.

(Female, 19 years old, PWP-MPU01-060220)

#### 3.1.2. Ethnicity and Culture

Choices and decisions to engage in maternal care were often based on strong traditional customs and practices. A traditional healer commented that she protected a woman in pregnancy by:

“…*giving them the rope to wear around their waist. I take that rope and I prepare it using traditional medicines before I give it to the person… So, we offer them means to protect themselves and their pregnancy*”.

(KII-THP02-15012019)

Other pregnant women expressed their strong beliefs based on tradition about maternal care. Among group discussion participants, it was reported that negative sentiments emanate from strong cultural beliefs that ancestors are able to give better protection to the unborn child than maternal immunisation programmes. This was explored in group discussions with both young and older pregnant women:

“…*The clinic does not help with anything, how does it protect the baby, you will then find yourself staying at home and not going to the clinic and have faith that your ancestors will protect the baby*”.

(Pregnant woman, 24 years old, FGD-PWP-HLU01-171219)

Health workers illustrated negative perceptions of pregnant women regarding the use of maternal vaccination and traditional medicine simultaneously:

“… *as I am saying they are (people) who use “isihlambezo” who believe that if they get this… this vaccination will affect the functioning of this “isihlambezo”. But as long as they believe in that thing (isihlambezo) you won’t do anything to them.… she won’t take a thing you are saying*…”.

(Female Advanced Midwifery healthcare worker, KII-A&MS-MPUK01-13022020)

Views were also expressed by the participants that younger women use traditional medicine because this is what their own mothers did:

“…*a woman has that belief, saying that no I’m not going anywhere for a period of time… you see, you will find that they do not believe in using the clinic. You see I grew up at home, my mother was not using the clinic, she was that kind of a person. They were using traditional medicines and only that*…”.

(Mother of pregnant woman, 39 years old, PPW-05-29062020)

Traditional beliefs are entrenched in the community of uMkhanyakude. These are perpetuated by the custodians of the community. Traditional and faith healers are important and respected people in society; their beliefs resonate among the community. Traditional healers encourage women to go to the clinics for antenatal care but they believe that pregnant women should also be protected by traditional medicine. Traditional socio-cultural beliefs are valued, thereby negatively influencing the likelihood of engaging with maternal immunisation, which poses challenges to women because of the need to regularly attend a clinic. The findings show that traditional healers encourage certain norms and practices when a woman is pregnant. Traditional healers we spoke to accepted that maternal immunisation is essential; however, they believe that pregnant women should also be protected by placing certain ropes tied around their waists to protect the baby. Traditional healers believe such practices do not interfere with any sort of healthcare received from the clinic because it does not enter one’s bloodstream. 

Traditional healers interviewed further confirmed that pregnant women consult them for herbal concoctions which cleanse their digestive systems, assist in labour and ensure good health for their unborn babies.

### 3.2. Cues to Action

#### 3.2.1. Family and Friends’ Pressure to Conform

Even when pregnant women are prepared to seek maternal care and immunisation, negative sentiments from family members influence them to discontinue care. The negative sentiments from family members mainly emanate from cultural beliefs. This was discussed in the focus group discussion, where the power of the ancestors was stressed.

“…*In other households you’d find that a pregnant woman may be allowed to go to the clinic maybe for the first day, but when she gets home her mother may change and say neither of my children attends the clinic, in this household we have ancestors*…”.

(Pregnant woman, 24 years old, FGD-PWP-HLU01-171219)

#### 3.2.2. Health Facility Regulations on Maternal Immunisation

Some participants perceived that health workers do not give any explanation when administering the maternal immunisation injection; therefore, it seems that some of the women considered that they were forced to engage without information. In this way, women have illustrated that they have accepted engagement with maternal immunisation because it is a requirement stated by the law.

“…*I did get it, isn’t it they vaccinated me without knowledge of what the vaccine is or (laughs). What can I say like if they are vaccinating us by force and hey it is the law, isn’t it we are ruled by the law*…”.

(Mother of pregnant woman, 39 years old, MPW-04-19062020)

### 3.3. Likelihood of Action

#### Barriers to Action

We use “barriers to action” to explain structural variables in health service delivery that inhibit engagement in maternal immunisation. The negative aspects of the health service delivery system act as impediments in efforts towards engagement. In this analysis, institutional barriers including low-quality health service delivery, long queues, distance to the health facilities, and immunisation vaccine stockouts, coupled with low levels of maternal knowledge, were identified as some barriers in engagement with maternal care.

Participants highlighted poor attitudes of some healthcare workers towards health service users.

“…*No, sometimes you come across nurses who have a bad attitude…I was reluctant to ask as to why are we being injected, what are we injecting for on the arm because all of us who were there they gave us that injection*”.

(Pregnant woman, 34 years old, IMPR-MPW-01-17062020)

Regarding the long queues at the clinics, participants highlighted that priority is mostly given to patients that come for delivery, causing long waiting times at health facilities. 

Health workers stated that pregnant women face challenges in accessing health facilities because of high transport costs and long distances to the clinic. Such long distances to the health facility require the use of motor transport, which tends to be costly for most women. Health workers stated that the lack of transport to the clinics has resulted in most pregnant women not meeting the required appointments.

“…*Eh I think others have the problem of their transportation. For the person to go to the clinic, that can have an impact of ended up not going*…”.

(Female Antenatal and Maternal healthcare worker, IMPR-A&MS-01-170120)

The stockouts of the immunisation vaccine have led pregnant women to miss relevant immunisation opportunities. This has resulted in some pregnant women receiving the tetanus vaccine only once, and later in gestation than recommended. Health workers complained about stockouts:

“…*maybe you find (out) that eh, there is no (more) vaccinations at the clinic, probably the vaccination will arrive at some other time*…”.

(Female Antenatal and Maternal healthcare worker, IMPR-KII-A&MS-01-13022020)

Some health workers suggested that some pregnant women lacked knowledge regarding maternal immunisation and the value of antenatal care, so some started to attend the clinic late or did not come until they were in labour. Some of the participants also highlighted that some women do not have maternal immunisation knowledge, and therefore they are puzzled about this practice in the clinic:

“…*It was my first time to get an injection on the arm, I even told them here at home that now when you start clinic you get injection, we were discussing as mothers as to why we are being injected now on the arm…. But I would not have had information as to what they were injecting us for*…”.

(Female, 34 years old, IMPR-MPW-01-17062020)

Our findings show that health behaviours and perceptions towards engaging with maternal vaccination are greatly influenced by perceived social norms, barriers to action and cues to action. We have shown that the acceptability of maternal immunisation depends on several external influences.

## 4. Discussion

The modified HBM theoretical framework was useful in illuminating several factors that may have negatively influenced the acceptance and uptake of maternal immunisation in uMkhanyakude. Pregnant women tend to delay in taking up maternal immunisation or entirely fail to attend the clinic for their immunisation. Cultural beliefs and practices are modifying factors that had a negative effect on maternal immunisation. People expressed indifference towards a vaccine because of poor knowledge [23]. 

Our findings suggest that health workers might not be taking time to give enough information about maternal immunisation, its intended purpose, and the actual diseases that pregnant women are susceptible to. A systematic review carried out globally, on factors influencing vaccine decision making among pregnant women, has shown that pregnant women who received a recommendation from a healthcare professional were more likely to receive a vaccination [24], underlining the importance of taking time to explain immunisation and support uptake among pregnant women. 

More education is needed about the diseases that pregnant women are susceptible to, so that individuals place higher value on the maternal vaccination. Studies have shown there is a strong correlation between maternal literacy and the uptake of vaccination [14,15], suggesting more effort may be required to reach women with lower literacy levels. To increase knowledge of maternal immunisation, antenatal and maternity staff, who are the trusted source of information, need to be trained to provide adequate information regarding maternal immunisation [24].

The acceptability of maternal immunisation is greatly influenced by user experiences when engaging with care. From the findings, health service delivery challenges have been experienced in this rural community, leading to the interrupted delivery of the maternal vaccination. For example, long waiting times at clinics may lead to dissatisfaction with the service which could lead to missed appointments [25]. This has been experienced in other studies across sub-Saharan Africa [5,10,11]. The major barriers of the health system are due to inadequate financial resources to secure sufficient stock of the vaccination translating to the inadequate delivery of vaccination services [11]. 

A lack of perceived family and/or peer approval of vaccinations during pregnancy is an important barrier to vaccine uptake [23]. In this analysis, pregnant women are influenced by both traditional healers and family. Upholding traditional beliefs about the protection provided by ancestors, for example, contributes to women declining immunisation in our study setting.

Given these findings, we agree with Shen et al. (2014), that sustained investments in routine vaccination, education campaigns, health systems responsiveness, and efficient service delivery are needed [26]. Although a substantial proportion of women attend antenatal care (ANC) during pregnancy in South Africa, there remain worrying gaps in access to ANC services and coverage [27,28]. Women in this study gave many reasons for not attending ANC and receiving maternal immunisation vaccines: the influence of traditional social norms, high transport costs and poor quality of care from health providers. Addressing these barriers through policies and frontline healthcare quality improvement measures can improve the appeal of maternal immunisation vaccines to expectant mothers [29]. Further analyses of maternal immunisation specific barriers and the means of addressing them are required to strengthen the existing programs and provide a more efficient delivery system for additional/other maternal vaccines. 

The importance vaccinating adults, including pregnant women, has been highlighted by the COVID-19 pandemic. COVID-19 vaccination has been recommended for pregnant women in many countries, including South Africa. The positive media coverage that vaccination, including maternal vaccination has had during the pandemic, may improve the knowledge and acceptability of maternal vaccination globally. Healthcare workers should be trained to inform pregnant women about maternal immunisation at each ANC visit. Information leaflets detailing procedures that are conducted at each ANC visit should be made available to pregnant women as paper copies, and shared on digital platforms, as well as provided through local radio programmes to reach less-literate women.

The study design has some limitations which may have influenced the results. For example, the participants of this study were from a rural setting, with poor backgrounds and limited education which could lead to bias in sample recruitment and the responses we were given. There was a likelihood of generalizing the results because the study sample was small. More views from the community could have been represented by a larger sample. This study applied the Health Belief Model as our organising framework for analysis, which emphasises individual level factors; therefore, other external determinants such as social/structural factors may not have been thoroughly covered in the findings given this focus.

## 5. Conclusions

Maternal immunisation uptake is greatly influenced by traditional customs and health service delivery challenges. Using a modified HBM model facilitated the highlighting of the impact that normative influences have on people’s perceptions and in turn their actions. These aspects influence the risk perceptions that an individual holds, thus impacting on the decision of whether or not to adhere to an immunisation regime. The strength of cues to action are important as they may have the potential to either positively or negatively influence a pregnant mother to either consider taking the immunisation or refusing to take it up. Understanding health-related behaviours and their influences and addressing barriers that block access can facilitate engagement with care.

## Figures and Tables

**Table 1:** Study participant description.

Study Participants	Number Interviewed Including the Focus Group Discussion	Description
Pregnant women	9	Pregnant women (Primigravida and Multipls)
Caregivers/mothers of pregnant women	7	Individuals who cared for pregnant women
Healthcare workers	4	Healthcare personnel specialising in advanced midwifery and as breastfeeding consultants
Traditional Healers	3	Traditional medicine practitioners
Faith healers	2	Individuals that were described as anointed for healing either traditional or spiritual
Church leaders	2	Religious leaders in the church
Community Midwife	1	Individuals who assist in childbirth within the community. Not registered with the Department of Health
Total	28	

## Data Availability

The datasets generated and/or analysed during the current study are not publicly available due to confidentiality assured to our participants ensuring that we would protect their anonymity but data are available from the corresponding author on reasonable request.

## Supplementary Materials

The following are available online at https://www.mdpi.com/article/10.3390/vaccines10030415/s1, S1: Antenatal & Maternity Staff topic guide, S2: Community leaders topic guide, S3: FGDs_Husbands and Mothers of Pregnant Women, S4: Pregnant Women topic guide, S5: Women in enrolled in maternal immunization trials topic guide.
