# Supplementary material for: Assessing Community Acceptance of Maternal Immunisation in Rural KwaZulu-Natal, South Africa: A Qualitative Investigation
Source: Vaccines (Basel). 2022 Mar 10;10(3):415. doi: 10.3390/vaccines10030415 (PMC8951159; doi:10.3390/vaccines10030415)
Supplement: Supplementary file 1 [file vaccines-10-00415-s001.zip › S4. Pregnant Women topic guide.pdf]

# Assessing community acceptancy and health facility preparedness for implementation of maternal immunisation programs in urban and rural South Africa

**Focus Group Discussions Topic Guide**

**Respondent groups:** pregnant mothers

**Introduction**

Good day, my name is………… And my colleagues are…………, and……… We are working at Africa Health Research Institute.

We would like you to participate in the study entitled: Assessing community acceptancy and health facility preparedness for implementation of maternal immunisation programs in urban and rural South Africa.

This study aims to explore community acceptancy and health facility preparedness for implementation of maternal immunisation programs in selected urban and rural settings in South Africa.

---

**Socio-Demographic Information of respondent (information to be collected for individual participant of the FGD)**

1.1. Age (years):____

1.2. Race:
__ White
__ Indian
__ Black
__ Coloured
__Other (specify) ________________________

1.3.Language group:
__ Tswana
__ Zulu
__ Xhosa
__ Tsonga
__ Venda
__ Swazi
__ Ndebele

__ Sotho
__ Pedi
__ Other (Specify)________________________

1.4. What is your preferred language?
__ English
__ Afrikaans
__ Other (specify)___________________

1.5. Highest level of education completed
__ Primary education
__ Some high school but didn't complete
__ Further Education Training (FET)
__ Grade 12\Matric
__ Some high school but didn't complete
__ Did not finish tertiary
__ Tertiary
__ College
__ Technical College
__ University
__Did not finish tertiary
__ Further Education Training (FET)
__ University Graduate

1.6. Current employment status
__ Employed full-time
__ Employed part-time
__ Unemployed
__ Volunteer work
__ Other (specify) ___________________________________

1.7. Occupation ________________________________________

1.8. Have you ever had children?
__Yes
__No

1.9. Do you have any children who are less than 5 years?
__ Yes
__ No

1.10. If yes, how many?____

**Part 1: Perceptions on maternal immunization**

1. What do you understand when you hear the term 'Maternal Immunisation'?

2. Please explain if you know of any immunisations that are currently given to pregnant women as part of routine care
    a. Probe: which vaccines do you think are given to pregnant women and why?
3. Did you receive any of these vaccinations?
4. Who do you think maternal immunisation protects?
    a. Look for the following answers:
        i. Pregnant mother only
        ii. Unborn baby only
        iii. Newborn baby (up to ~3 months) only
5. If you could receive a vaccine during your pregnancy, would you accept it?
    a. Yes/ No- Please explain why or why not
    b. If a vaccine was available free of charge in clinic, would you accept it?
    c. If vaccine was available, but you had to pay for it, (<R150) would you pay for it, in order to protect you/ your baby?

**Part 2: Community perceptions on maternal immunization**

**1. Fears**

Do you have any fears regarding maternal immunization?

Please explain what your fears are and why do you have them?

**2. Views**

What are some of the common views that you hear in your community relating to maternal immunization?
Please explain why you think they hold such views

**3. Myths**

Are there any myths that are prevalent in your community relating to maternal immunization?

Please elaborate

4. **Misconceptions**

What are the misconceptions that you hear relating to maternal immunization?

Please explain

**Part 2:**
**Acceptability of maternal immunization**

1. **Social factors**
   i. Do women accept maternal immunisation more easily than men?
      a. Please explain.
   ii. Do younger people accept maternal immunization more easily than older people?
      a. Please explain
   iii. Do people who live in urban areas accept maternal immunisation more easily than people who live in rural areas?
      a. Please explain

2. **Cultural factors**

What are some of the cultural beliefs that could facilitate or impede acceptability of maternal immunization?

Please explain

3. **Religious factors**

What are some of the religious beliefs that could facilitate or impede acceptability of maternal immunization?

Please explain

4. **Economic factors**

What are some of the economic factors that could facilitate or impede acceptability of maternal immunization?

Please explain
