# Supplementary material for: Assessing Community Acceptance of Maternal Immunisation in Rural KwaZulu-Natal, South Africa: A Qualitative Investigation
Source: Vaccines (Basel). 2022 Mar 10;10(3):415. doi: 10.3390/vaccines10030415 (PMC8951159; doi:10.3390/vaccines10030415)
Supplement: Supplementary file 1 [file vaccines-10-00415-s001.zip › S5. Women in enrolled in maternal immunization trials topic guide.pdf]

Assessing community acceptancy and health facility preparedness for implementation of maternal immunisation programs in urban and rural South Africa

Semi-structured Interviews or Individual In-depth Interviews

Respondent groups: Women enrolled in maternal immunisation trials and women who previously had a child with Group B streptococcus sepsis (survived or died)

Introduction

Good day, my name is………… I am a……………at Africa Health Research Institute. I would like you to participate in the study entailed: Maternal immunisation in South Africa: exploring acceptancy and preparedness for implementation of maternal immunisation programs in urban and rural sites in South Africa.

This study aims to explore community acceptancy and health facility preparedness for implementation of maternal immunisation programs in selected urban and rural settings in South Africa.

Socio-Demographic Information of respondent

1.1. Age (years):____

1.2. Race:
__ White
__ Indian
__ Black
__ Coloured
__Other (specify) ________________________

1.3. Language group:
__ Tswana
__ Zulu
__ Xhosa
__ Tsonga
__ Venda
__ Swazi
__ Ndebele
__ Sotho

__ Pedi
__ Other (Specify)________________________

1.4. What is your preferred language?
__ English
__ Afrikaans
__ Other (specify)___________________

1.5. Highest level of education completed
__ Primary education
__ Some high school but didn't complete
__ Further Education Training (FET)
__ Grade 12\Matric
__ Some high school but didn't complete
__ Did not finish tertiary
__ Tertiary
__ College
__ Technical College
__ University
__Did not finish tertiary
__ Further Education Training (FET)
__ University Graduate

1.6. Current employment status
__ Employed full-time
__ Employed part-time
__ Unemployed
__ Volunteer work
__ Other (specify) ___________________________________

1.7. Occupation ____

1.8. Have you ever had children?
__Yes
__No

1.9. Do you have any children who are less than 5 years?
__ Yes
__ No

1.10. If yes, how many?____

Part 1: Community perceptions on maternal immunization

Maternal Immunisation

1. What do you understand when you hear the term 'Maternal Immunisation'?

2. Please explain if you know of any immunisations that are currently given to pregnant women as part of routine care
   a. Probe: which vaccines do you think are given to pregnant women and why?
3. Did you receive any of these vaccinations?
4. Who do you think maternal immunisation protects?
   a. Look for the following answers:
      i. Pregnant mother only
      ii. Unborn baby only
      iii. Newborn baby (up to ~3 months) only
5. If you could receive a vaccine during pregnancy, would you accept it?
   a. Yes/ No- Please explain why or why not
   b. If a vaccine was available free of charge in clinic, would you accept it?
   c. If vaccine was available, but you had to pay for it, (<R150) would you pay for it, in order to protect you/ your baby?
6. Fears
   a. In your community, do people have any fears or concerns regarding maternal immunization?
   b. Please explain your answer
7. Views
   a. What are some of the common views that you hear in your community relating to maternal immunization?
   b. Please explain why you think they hold such views
8. Beliefs
   a. Are there any beliefs that are prevalent in your community relating to maternal immunization?
   b. Please elaborate
9. Misconceptions

   What are the good and bad things that you hear relating to maternal immunization?

   Please explain

Part 2: Acceptability of maternal immunization

1. Social factors
   i. Do women accept maternal immunisation more easily than men?
      a. Please explain.
   ii. Do younger people accept maternal immunization more easily than older people?
      a. Please explain
   iii. Do people who live in urban areas accept maternal immunisation more easily than people who live in rural areas?
      a. Please explain
2. Cultural factors
   a. What are some of the cultural beliefs that could facilitate acceptability of maternal immunization?
      Please explain
   b. What are some of the cultural beliefs that could impede acceptability of maternal immunization?
      Please explain

3. Religious factors
   a. What are some of the religious beliefs that could facilitate acceptability of maternal immunization?
      Please explain
   b. What are some of the religious beliefs that could impede acceptability of maternal immunization?
      Please explain

4. Economic factors
   a. What are some of the economic factors that could acceptability of maternal immunization?
      Please explain
   b. What are some of the economic factors that could impede acceptability of maternal immunization?
      Please explain
